# Identification and Functional Analysis of a Novel MIP Gene Mutation Associated with Congenital Cataract in a Chinese Family

**DOI:** 10.1371/journal.pone.0126679

**Published:** 2015-05-06

**Authors:** Xingchao Shentu, Qi Miao, Xiajing Tang, Houfa Yin, Yingying Zhao

**Affiliations:** 1 Eye Center, Second Affiliated Hospital of Zhejiang University School of Medicine, Hangzhou, Zhejiang Province, China; 2 Zhejiang Provincial Key Laboratory of Ophthalmology, Hangzhou, Zhejiang Province, China; National Eye Institute, UNITED STATES

## Abstract

Congenital cataracts are major cause of visual impairment and blindness in children and previous studies have shown about 1/3 of non-syndromic congenital cataracts are inherited. Major intrinsic protein of the lens (*MIP*), also known as AQP0, plays a critical role in transparency and development of the lens. To date, more than 10 mutations in *MIP* have been linked to hereditary cataracts in humans. In this study, we investigated the genetic and functional defects underlying a four-generation Chinese family affected with congenital progressive cortical punctate cataract. Mutation screening of the candidate genes revealed a missense mutation at position 448 (c.448G>C) of *MIP*, which resulted in the substitution of a conserved aspartic acid with histidine at codon 150 (p.D150H). By linkage and haplotype analysis, we obtained positive multipoint logarithm of odds (LOD) scores at microsatellite markers *D12S1632* (Z_max_ = 1.804 at α = 1.000) and *D12S1691* (Z_max_ = 1.806 at α = 1.000), which flanked the candidate locus. The prediction results of PolyPhen-2 and SIFT indicated that the p.D150H mutation was likely to damage to the structure and function of AQP0. The wild type and p.D150H mutant AQP0 were expressed in HeLa cells separately and the immunofluorescence results showed that the WT-AQP0 distributed at the plasma membrane and in cytoplasm, while AQP0-D150H failed to reach the plasma membrane and was mainly retained in the Golgi apparatus. Moreover, protein levels of AQP0-D150H were significantly lower than those of wide type AQP0 in membrane-enriched lysates when the HEK-293T cells were transfected with the same amount of wild type and mutant plasmids individually. Taken together, our data suggest the p.D150H mutation is a novel disease-causing mutation in *MIP*, which leads to congenital progressive cortical punctate cataract by impairing the trafficking mechanism of AQP0.

## Introduction

According to WHO criteria, there are estimated 1.4 million children affected by irreversible blindness worldwide [1]. Childhood blindness is mainly caused by congenital cataracts, which severely impair the normal visual development of children. Previous reports have shown that about 1/3 of non-syndromic congenital cataracts are inherited [2]. Among these, autosomal dominant inheritance is the most prevalent. To date, there are at least 20 genes having been identified and mutations in these genes have been linked to autosomal dominant inherited cataracts. These include genes encoding crystallins, membrane proteins (such as gap junction protein alpha-3 [*GJA3*], gap junction protein alpha-8 [*GJA8*] and *MIP*), cytoskeletal proteins (beaded filament structural protein 2 [*BFSP2*]), and some growth and transcription factors (including heat shock transcription factor 4 [*HSF4*], Maf-like protein [*MAF*] and paired-like homeodomain 3 [*PITX3*]) [3].

Major intrinsic protein of the lens (*MIP*), known as AQP0, belongs to the superfamily of AQPs. It is expressed predominantly in mature lens fiber cells and accounts for approximate 44.8% of the total plasma membrane proteins [4]. Similar to other AQPs, AQP0 serves as a water channel which facilitates the movement of water and small neutral solutes across the plasma membrane [5]. In spite of low water permeability compared to AQP1, which expresses in the lens epithelial cells, AQP0 plays a crucial role in maintaining cellular water homeostasis in avascular lens. Homozygous or heterozygous loss of AQP0 will reduce the water permeability of lens fiber cells considerately and result in opacities of lens in mice [6]. In addition, AQP0 forms “thin junctions” between adjacent lens fibers in terms of adhesion molecule, which is also required for lens transparency. Lenses of AQP0-deficient mice lose ordered packing and display apparent fiber cell disorganization. Moreover, this leads to the formation of cataract although the impaired water permeability has been compensated by the transgenic AQP1 [7]. Besides the intermolecular contacts among AQP0 monomers, it also plays an important role in interaction with other proteins in lens fiber cells, such as crystallins [8] and connexins [9].

So far, 12 mutations in *MIP* have been identified and linked with autosomal dominant cataract. The different cataract phenotypes caused by these mutations indicate diverse functions of AQP0 in the lens, as well as complicated pathogenic mechanisms. Previous studies have shown that most mutations in AQP0 would impair its normal trafficking, causing the protein to retain within cytoplasm instead of locating at the plasma membrane. This would result in the loss of water channel function, which may contribute to the forming of cataract [10–12].

In this work, we studied on a four-generation Chinese family affected with congenital progressive cortical punctate cataract. We identified a novel missense mutation in exon 2 of *MIP*, which substituted histidine for aspartic acid (p.D150H). The mutant would damage the trafficking mechanism of AQP0, and decrease the amount of protein at the plasma membrane significantly both in HeLa and HEK-293T cells.

## Materials and Methods

### Ethics statement

The study was conducted in accordance with the tenets of the Declaration of Helsinki and approved by the ethics committee of 2nd Affiliated Hospital, Medical College of Zhejiang University, Hangzhou, China. Written informed consent was obtained from all participants or their guardians.

### Clinical evaluation and collection of DNA specimens

A four-generation Chinese family affected with autosomal dominant congenital cataract was recruited for the study at Eye Center of the 2nd Affiliated Hospital, Medical College of Zhejiang University. All participants underwent detailed ophthalmologic examinations including visual acuity, intraocular pressure, slit-lamp examination and fundus examination with the dilated pupils. The phenotypes were documented by slit-lamp photography. In addition, a total of 100 unrelated ethnically matched controls with no family history of congenital cataracts were recruited. Peripheral blood samples of all participants were collected in Vacutainer tubes (Becton-Dickinson, Franklin Lakes, NJ, USA) containing ethylene diamine tetraacetic acid (EDTA) and genomic DNA was extracted using the Simgen Blood DNA mini kit (Simgen, Hangzhou, China).

### Mutation screening

Mutation screening was performed in genomic DNA samples from affected and unaffected family members. Ten genes most frequently involved in autosomal dominant cataract were analyzed by directly sequencing: *CRYAA*, *CRYAB*, *CRYBA3/A1*, *CRYBB1*, *CRYBB2*, *CRYGC*, *CRYGD*, *MIP*, *GJA3* and *GJA8*. All exons and intron-exon junctions of the candidate genes were amplified by polymerase chain reaction (PCR) using previously published primer sequences [13]. The PCR products were isolated by electrophoresis on 1.0% agarose gels and sequenced with the BigDye Terminator Cycle sequencing kit V3.1 (Applied Biosystems, Foster City, CA) on an Applied Biosystems PRISM 3730 Sequence Analyzer. The sequencing results were analyzed using Chromas 2.33 and compared with the sequences in the NCBI GenBank database.

### Genotyping and linkage analysis

Fluorescently labeled microsatellite markers were used to corroborate the locus responsible for the disease. Microsatellite markers, allele frequencies and their distances were based on the Marshfield database and the UCSC database. Multipoint linkage analysis and reconstruction of the most likely haplotypes were performed using the linkage program MERLIN (http://www.sph.umich.edu/csg/abecasis/Merlin/index.html). The disease was modeling as an autosomal dominant trait with a disease allele frequency equal to 0.0001. For calculating logarithm of odds (LOD) scores, the disease penetrance was assumed to be 80%, 90%, 99% and 100% respectively.

### Bioinformatics analysis

To predict the effect of this amino acid substitution on the protein, we used the online tools including PolyPhen-2 (Polymorphism Phenotyping, http://genetics.bwh.harvard.edu/pph2/) and SIFT (Sorting Intolerant Form Tolerant, http://sift.jcvi.org/) programs. Using structural and comparative evolutionary considerations, the prediction result of PolyPhen-2 is one of the following: benign, possibly damaging and probably damaging. While the SIFT is based on evolutionary conservation and the outcome ranges from 0 to 1. The amino acid substitution is predicted damaging if the score is < = 0.05, and tolerated if the score is > 0.05.

### Construction of plasmids which encode WT-AQP0 or AQP0-D150H

The coding sequence of wild type human AQP0 was amplified by PCR with the following primers: sense primer 5’-TCGACTCGAGATGTGGGAACTGCGATCA-3’; antisense primer 5’-TCGAAAGCTTAGCTGGAGCTTCTACAGG-3’.Then the PCR products were gel purified and cloned into the eukaryotic expression vector, pcDNA3.1^-^ (Invitrogen, Carlsbad, CA, USA). The plasmid expressing AQP0-D150H was obtained by using site-directed mutagenesis with the following primers: sense primer 5’- CATCTTTGCCACATACCACGAGAGGCGGAATGG-3’; antisense primer 5’- CAGAGCACGAACTGGAGCGTCAGGAAGATCTCC-3’. Both wild type and mutant vectors were confirmed through directly sequencing.

### Cell culture and transfection

HeLa and HEK-293T cells were cultured in RPMI-1640 medium supplemented with 10% fetal bovine serum (FBS) in a 37°C incubator with 5% CO_2_. Transient transfections of wild type and mutant plasmids were carried out seperately using Lipofectamine 2000 reagents (Invitrogen, Carlsbad, CA, USA) according to the manufacturer’s protocols.

### Immunofluorescence microscopy for subcellular location

HeLa cells were seeded on cover slips in 6-well plates one day before transfection at approximately 60% confluency. Twenty four hours after transfection, cells were washed with PBS and fixed with 4% paraformaldehyde in PBS for 10 min at room temperature. After rinsing with PBS and blocking with donkey serum, cells were subjected to immunofluorescence using mouse monoclonal anti-AQP0 antibodies (Santa Cruz Biotechnology, CA, USA) and rabbit monoclonal anti-GM130 antibodies (Abcam, Cambridge, UK) and AlexaFluor488-conjugated donkey anti-mouse and AlexaFluor555-conjugated donkey anti-rabbit IgG antibodies (Life Technologies, Carlsbad, CA, USA). Cells were further incubated with Hoechst33258 to stain nuclei. Specimens were observed and images were captured using an Olympus FluoView 1000 confocal microscope with a 60×oil immersion objective.

### Western blotting analysis

After transfected with wild type and mutant plasmids separately, HEK-293T cells were lysed and membrane proteins were extracting with Mem-PER Plus Membrane Protein Extraction Kit (Thermo, USA) following manufacturer’s protocol. Same amount of membrane-enriched lysates were loaded in each individual gel lane, separated by 12% SDS-PAGE and transferred to polyvinylidene difluoride (PVDF) membranes. The membranes were immunoblotted with antibodies of anti-AQP0 (Santa Cruz Biotechnology, CA, USA) and anti-GAPDH (Cell Signaling Technology, Inc, USA). The signals were detected using an enhanced chemiluminescence(ECL) system (Pierce, Biotechnology Inc., Rockford, USA).

## Results

### Clinical data

**Fig 1:**
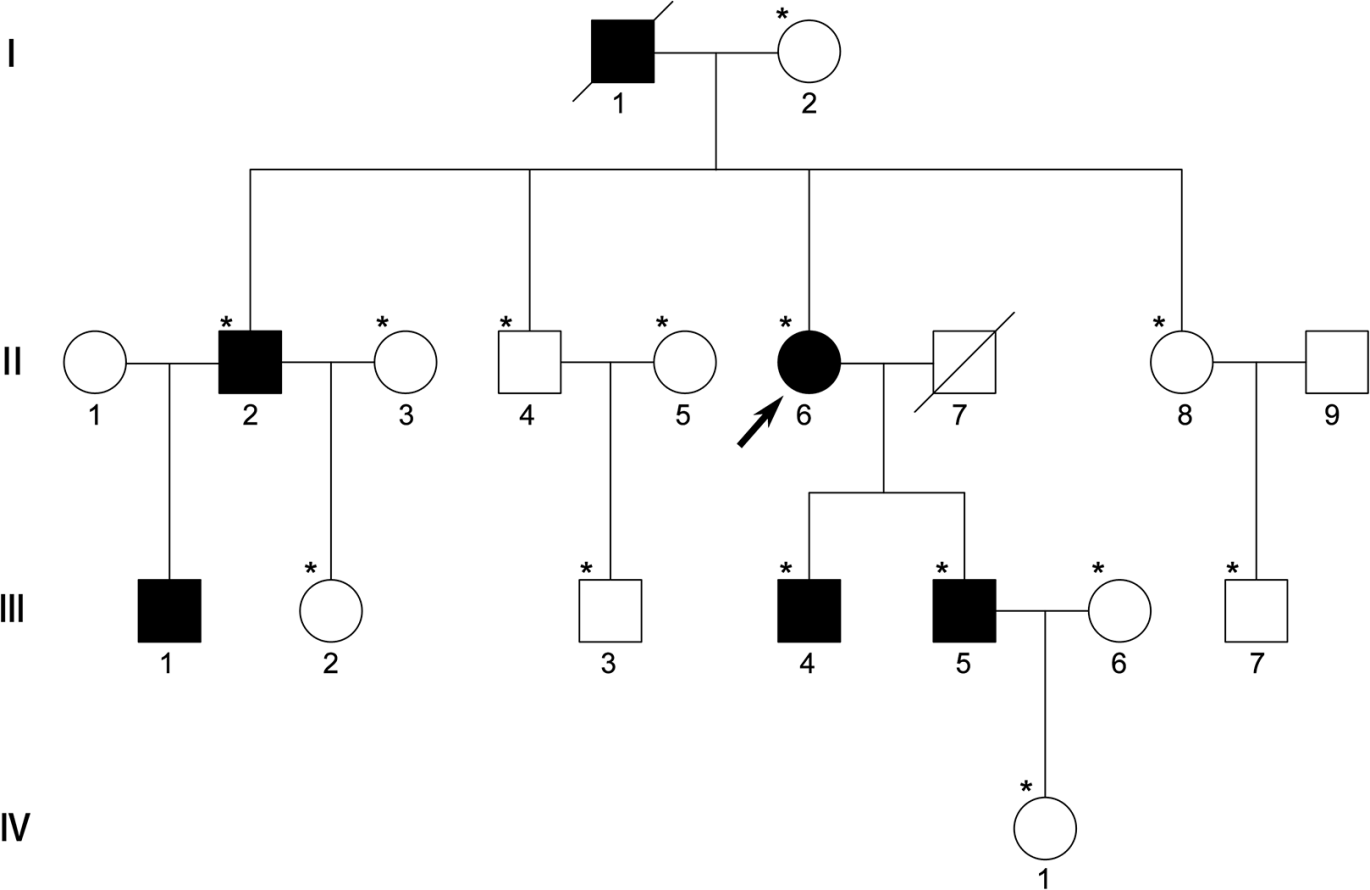
Family pedigree. A four-generation Chinese family affected with autosomal dominant cataract is shown. Squares and circles indicate males and females, respectively. The black symbols represent the affected members and open symbols represent the unaffected individuals. The diagonal line indicates a deceased family member and the black arrow indicates the proband. The family members attending this study are marked with asterisks.

**Fig 2:**
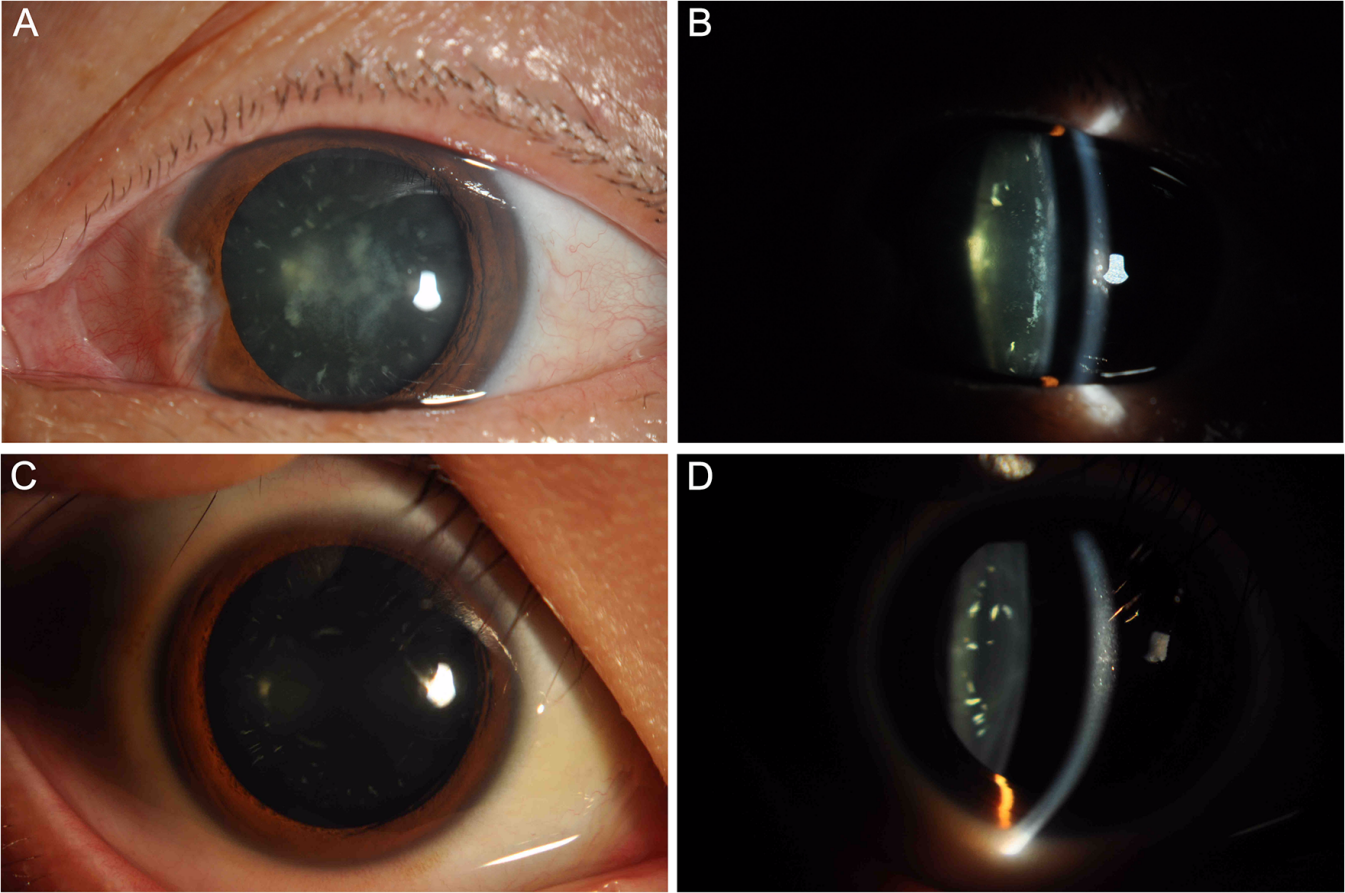
Clinical features of the proband (A, B) and her son (C, D). Slit-lamp photographs (diffuse illumination and silt lamp) show the phenotype of the congenital cataract is punctate cortical opacities. In younger affected one, the punctate opacities were only seen in the peripheral cortex of the lens. The punctate opacities became denser and invaded into central cortex of the lens gradually with increasing age.

We identified a four-generation Chinese family (6 affected and 13 unaffected) with a diagnosis of congenital progressive cortical punctate cataract. The pedigree of the family (Fig 1) revealed an autosomal dominant inheritance pattern for them. The proband was a 56-year-old female with a complaint of blur vision of both eyes (visual acuity was 0.25 in both eyes). Her elder brother (Ⅱ.2) and nephew (Ⅲ.1) also suffered from congenital cataract and had undergone cataract surgery several years ago. However, neither of the proband’s sons (Ⅲ.4,Ⅲ.5) complained of significant visual deterioration except for dazzle during driving at night. As shown in Fig 2A and 2B, the phenotype of congenital cataract was punctate cortical opacities. In younger affected one, the punctate opacities were only seen in the peripheral cortex of the lens (Fig 2C and 2D). The punctate opacities increased in number and became denser gradually with increasing age. Thus the proband presented central opacity as well as peripheral punctate opacities. There were no other ocular or systemic abnormalities in all affected members.

### Mutation analysis

**Fig 3:**
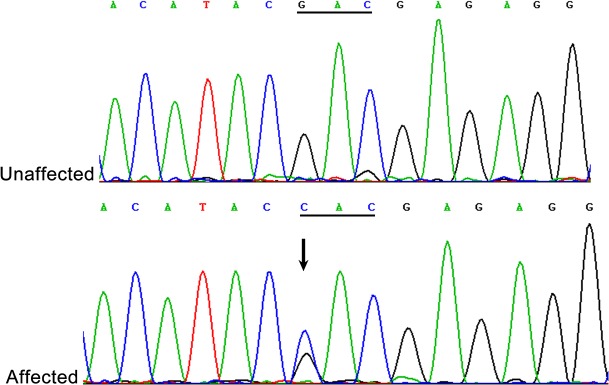
Mutation screening. Forward sequence analysis of exon 2 of MIP in the normal and affected members of this family. The DNA sequence chromatogram shows a heterozygous G>C nucleotide change (black arrow) in exon 2 of MIP (c.448G>C), which leads to the replacement of aspartic acid (GAC) with histidine (CAC) at codon 150 (p.D150H).

The direct sequencing of candidate genes revealed a heterozygous change (G>C),at position 448 (c.448G>C) of *MIP*, which leads to the replacement of a conserved aspartic acid with histidine at codon 150 (p.D150H) (Fig 3). This change was cosegregated with all affected individuals and not observed in other unaffected family members or 100 unrelated normal controls. No variant was found in exons of *CRYAA*, *CRYAB*, *CRYBA3/A1*, *CRYBB1*, *CRYBB2*, *CRYGC*, *CRYGD*, *GJA3* and *GJA8* genes.

### Linkage and haplotype analysis

**Fig 4:**
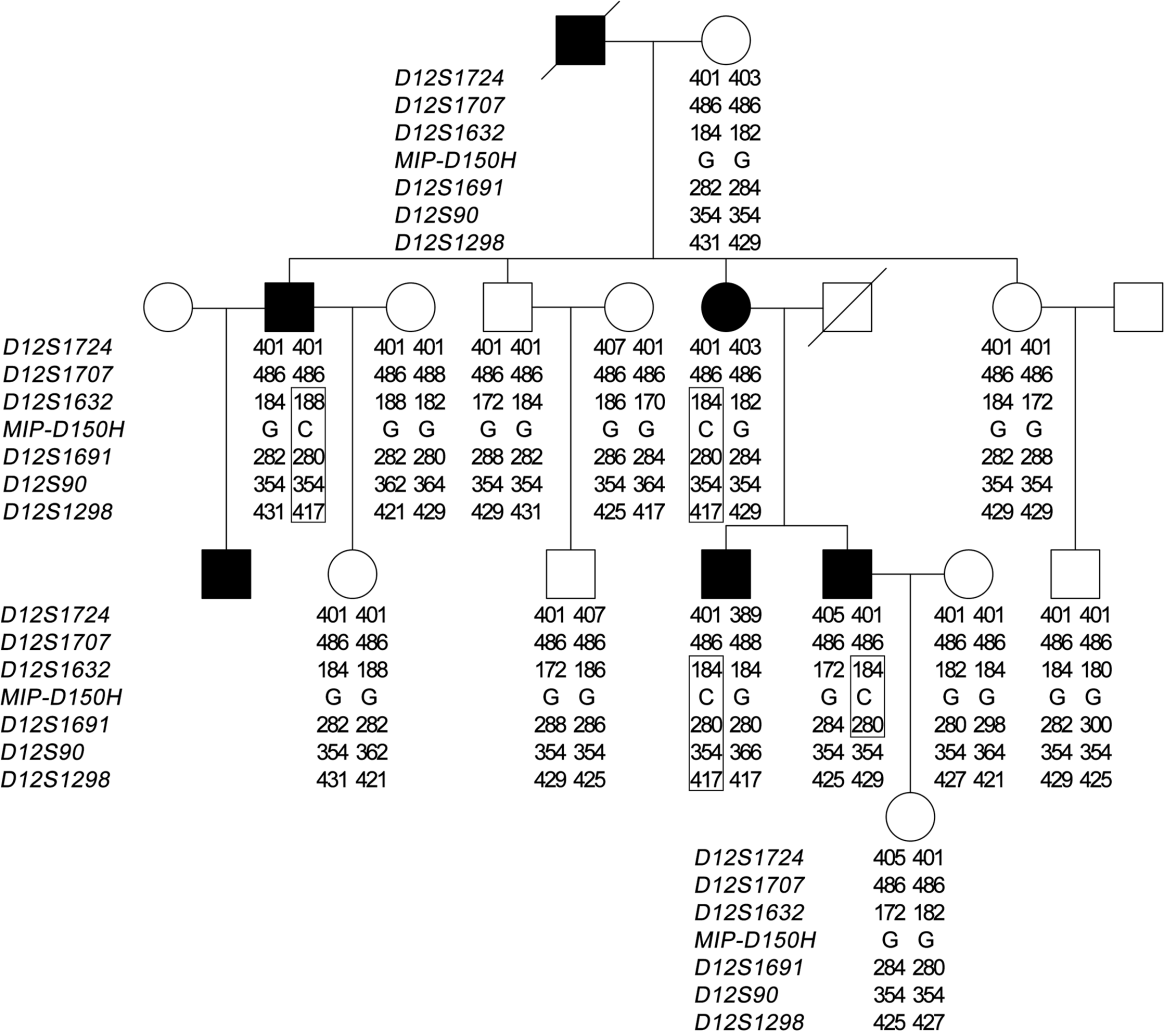
Haplotype of the cataractous family. Eight locus around *MIP* were genotyped. The disease-susceptibility haplotype (indicated by a vertical box) showed cosegregation with affected members in this family from *D12S1632* to *D12S1691*.

Twelve members of the affected family, including 4 affected individuals and 9 unaffected individuals were genotyped and studied by linkage analysis. For chromosome 12q13, around the *MIP* locus, seven locus genotypes and inferred haplotypes were showed (Fig 4) and multi-point LOD scores were summarized (Table 1). Positive multipoint LOD scores were obtained at markers *D12S1632* and *D12S1691* with the maximum LOD score approaching 1.806 at marker *D12S1691* (α = 1.000). The haplotype analysis revealed complete cosegregation in affected members.

**Table 1:** Multipoint LOD scores for linkage analysis between cataract locus and markers on 12q13.

Marker	Marshfield, cM	LOD scores by the disease penetrance			
		0.800	0.900	0.990	1.000
*D12S1724*	69.82	1.596	1.684	1.762	1.771
*D12S1707*	69.82	1.600	1.688	1.766	1.774
*D12S1632*	71.61	1.627	1.716	1.796	1.804
*MIP*: c.448G>C, p.D150H		1.628	1.718	1.797	1.806
*D12S1691*	72.2	1.628	1.718	1.797	1.806
*D12S90*	71.61	1.331	1.421	1.500	1.509
*D12S1298*	75.17	-2.207	-2.198	-2.194	-2.194

### Bioinformatics analysis

**Fig 5:**
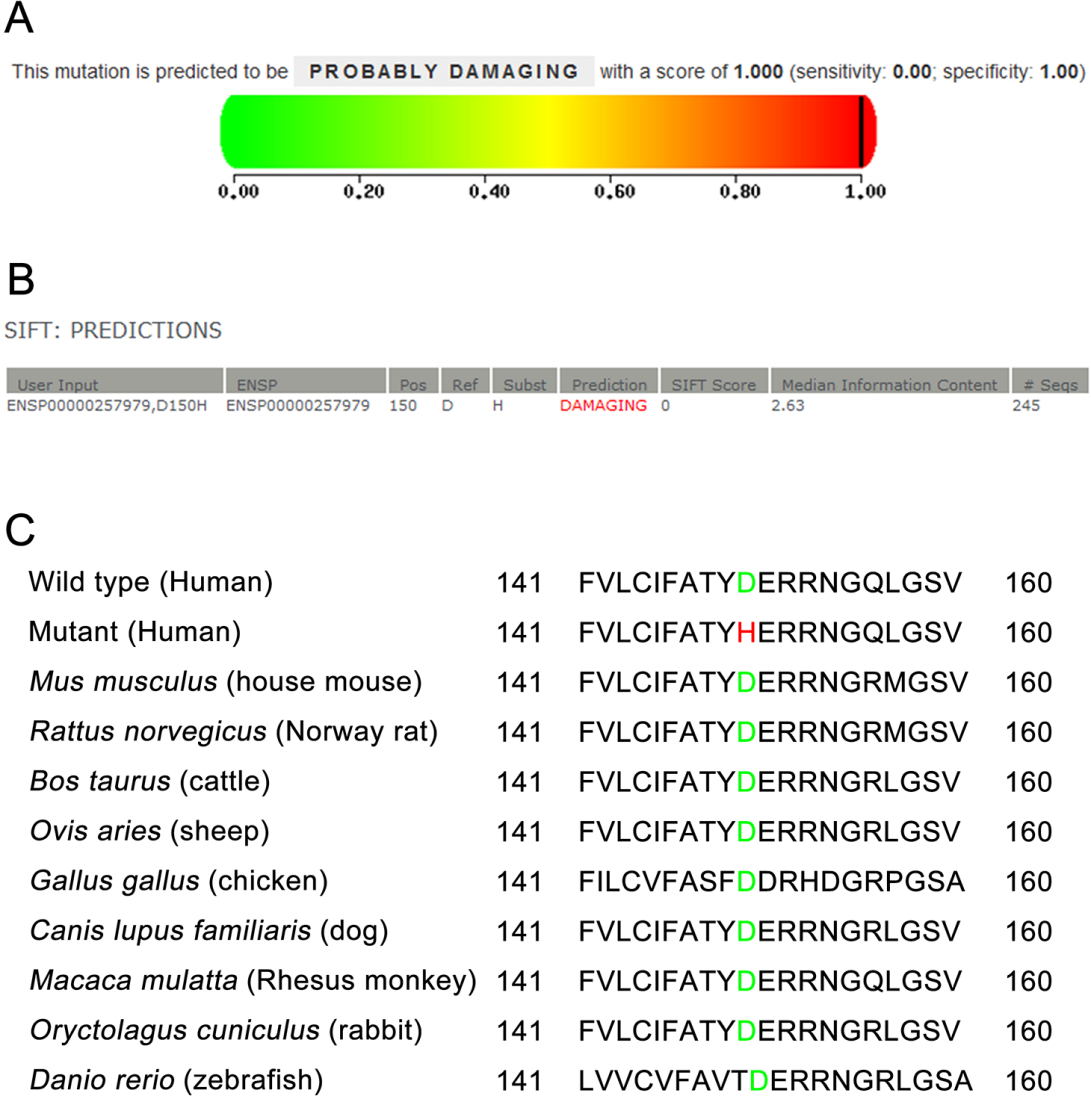
Bioinformatics analysis of the p.D150H mutation and multiple sequence alignment. (A) The mutation was predicted to be probably damaging with a score of 1.00 by PolyPhen-2, which meant it may have deleterious effect on the structure and function of the protein. (B) the score and media information content from SIFT was 0.00 and 2.63 respectively, predicting the effect of this amino acid substitution on protein function was damaging. (C) The result of a multiple sequence alignment from various species showed that the aspartic acid at position 150 of AQP0 is highly conserved (marked in green, the mutation in red).

In order to evaluate the impact of p.D150H mutation on the structure and function of AQP0, the PolyPhen-2 and SIFT were used. The mutation was predicted to be probably damaging with a score of 1.00 by PolyPhen-2 (Fig 5A), which meant it may have deleterious effect on the structure and function of the protein. Similarly, the score and media information content from SIFT was 0.00 and 2.63 respectively (Fig 5B), predicting the effect of this amino acid substitution on protein function was damaging. These results together indicated p.D150H mutation is likely to be deleterious to the protein and responsible for this congenital cataract.

### Multiple sequence alignment

The result of a multiple sequence alignment by BLAST showed that the aspartic acid at position 150 of AQP0 (*Homo sapiens*, NP_036196.1) is highly conserved among various species, including *Mus musculus* (NP_032626.2), *Rattus norveqicus* (NP_001099189.1), *Bos taurus* (NP_776362.1), *Ovis aries* (NP_001153230.1), *Gallus gallus* (NP_989597.1), *Canis lupus families* (NP_001074369.1), *Macaca mulatta* (XP_001115118.1), *Oryctolaqus cuniculus* (NP_001093431.1) and *Danio rerio* (NP_001003534.1) (Fig 5C).

### Subcellular location of WT-AQP0 and AQP0-D150H

**Fig 6:**
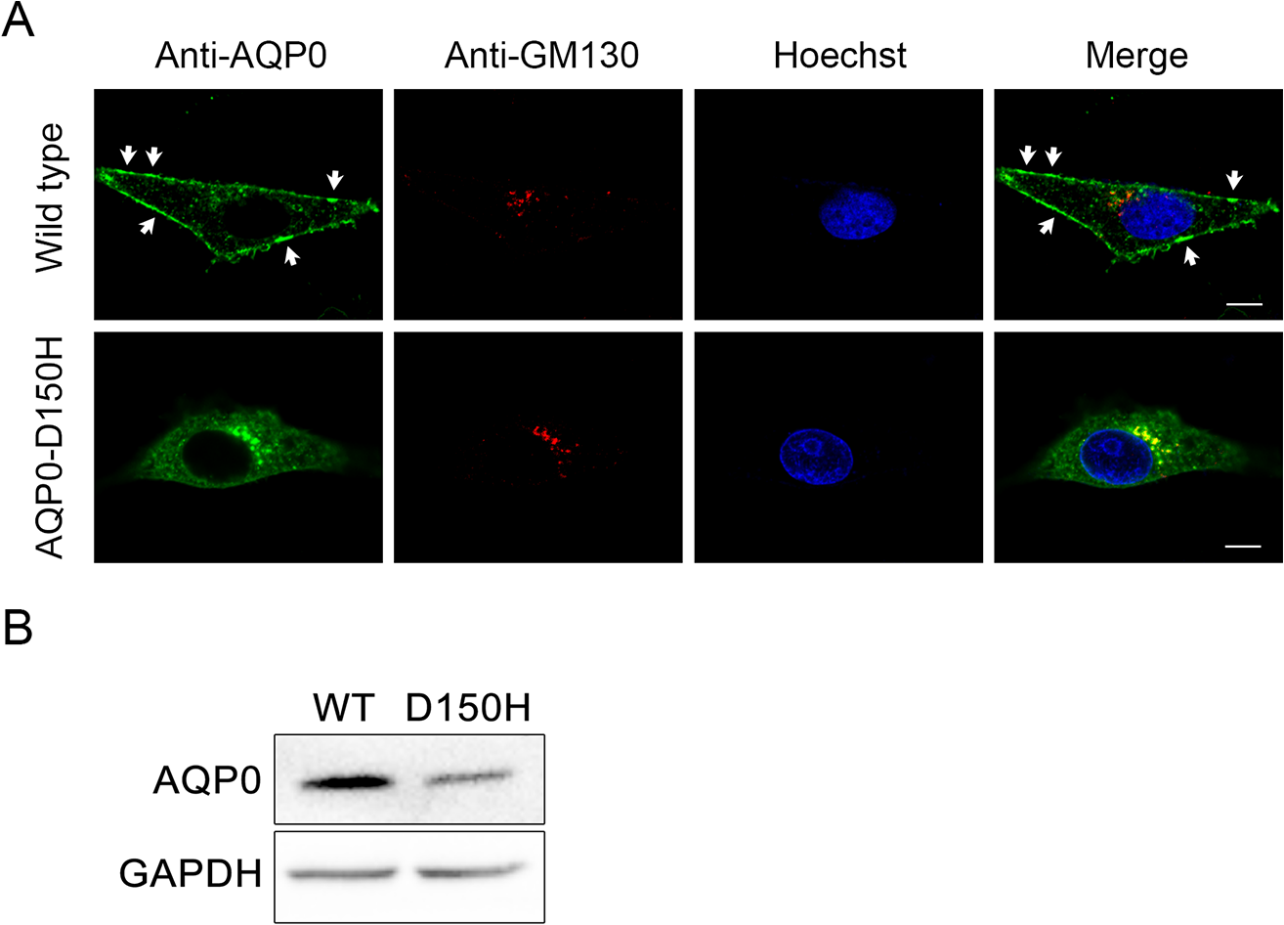
Subcellular location of WT-AQP0 and AQP0-D150H in two expressing cells. (A) Representative fluorescence microscopy images show the distributions of immunoreactive AQP0 and a Golgi apparatus resident protein (GM130) in HeLa cells which were transiently transfected with wild type AQP0 or AQP0-D150H. The wild type AQP0 was detected mainly at the plasma membrane (white arrow) and in cytoplasm. By contrast, AQP0-D150H was not observed at the plasma membrane other than cytoplasmic sites which extensively overlapped with that of GM130. Scale bar = 10μm. (B) The quantities of wild type and p.D150H mutant AQP0 in membrane-enriched lysates of HEK-293T cells were assessed by western blotting, after WT-AQP0 or AQP0-D150H transfected, GAPDH was used as control.

To investigate whether the p.D150H mutation impacts the normal trafficking of AQP0, immunofluorescence was performed after transient transfection of wild type and mutant AQP0 into HeLa cells individually. As expected, the wild type AQP0 was detected mainly at the plasma membrane and in cytoplasm, which is consistent with the normal cellular distribution of a plasma membrane protein. By contrast, AQP0-D150H was not observed at the plasma membrane other than cytoplasmic sites which extensively overlapped with that of GM130, a protein that localizes within Golgi apparatus (Fig 6A). Furthermore, as shown in Fig 6B, levels of AQP0-D150H were lower than levels of wide type AQP0 in membrane-enriched lysates when the HEK-293T cells were transfected with the same amount of wild type and mutant plasmids individually. These data demonstrate that the p.D150H mutation would damage the trafficking mechanism of AQP0 and result in the retention of protein within cytoplasm.

## Discussion

In this study, we investigated the genetic and functional defects underlying a four-generation Chinese family affected with congenital progressive cortical punctate cataract. Through direct sequencing the candidate genes, we identified a mutation in *MIP*, a G>C heterozygous transversion at position 448, which was predicted to replace the conserved aspartic acid with a histidine (p.D150H) in AQP0. This mutation was cosegregated with the phenotype in the family and not found in 100 unrelated normal controls. Moreover, we obtained positive multipoint LOD scores with microsatellite markers flanking the *MIP* gene and these might be near the maximum LOD score that could be generated by this family as cosegregation with the mutation and surrounding markers was complete, although the linkage and haplotype analysis were limited by the size of the pedigree. As a result, we identified this novel mutation of AQP0 (p.D150H) as the genetic defect of this congenital cataract family. To our knowledge, this is the first mutation in the intracellular loop D of AQP0 being associated with congenital cataract. Till now, twelve mutations of *MIP* have been reported to be associated with the autosomal dominant congenital cataract in humans (p.M1T [14], p.R33C [15], p.R113X [13], p.E134G [16], p.T138R [16], p.G165D [12], p.Y177C [17], p.R187C [18], p.V203fs [19], p.G213VfsX46 [20], p.G215D [21] and p.R233K [22]). As the presence of clinical heterogeneity of hereditary cataracts, the relationship between genotype and phenotype is complicated and undetermined, which means the same mutation in different families or within the same family can result in radically different cataract morphologies and severities, and vice versa [23]. Thus it is not surprising that the cataract phenotypes varied among *MIP* mutation families. The clinical features of younger affected members in our family are similar to a cataract family reported by Ding et al [21]: both characterized as punctate opacities in the cortex. In our family, the opacities became denser and invaded into central cortex of the lens with age. This may explain the different major complaints among the affected members, especially between younger and elder members.

AQP0 is the predominant protein within lens fiber cell membranes and plays important roles in homeostasis and transparency of the lens. As a membrane protein, AQP0 inserts in the plasma membrane with six transmembrane domains, resulting in six transmembrane helices, three extracellular loops, two intracellular loops, and the NH2- and COOH-terminal intracellular domains [5]. Structural and simulation studies have suggested the specific domain is required for the distinct function of AQP0 [24, 25]. Mutations in these domains disturb the physiological functions of AQP0 in the lens and contribute to the forming of cataracts. Despite a number of disease-causing mutations identified, only a few of them have been functional characterized. The mutations in transmembrane domains, such as p.E134G, p.T138R, p.G165D and p.G215D, show loss of water permeability due to impaired trafficking of the mutant proteins in heterologous expression systems [12, 16, 21]. In addition, the accumulation of unfolded protein in the endoplasmic reticulum (ER) caused by p.G213VfsX46 mutation increases the ER stress and results in cell death due to necrosis [11]. These results, along with previous structural studies, demonstrate that the membrane-spanning helices of AQP0 are crucial for the peptide to fold and reach the plasma membrane. Similarly, the AQP0 transgenic mouse models also attribute the congenital cataracts to the lowering water permeability and abolishing trafficking [26]. In contrast, the cataract-linked p.R33C mutation, which locates at the extracellular loop A of AQP0, poses no damage to the protein location or water permeability but causes reduction of cell-to-cell adhesion [27]. Both the in vivo and in vitro experiments indicate the adhesion property of AQP0 is essential in maintaining the structure and transparency of the lens [7, 28].

Since there is no previous study revealing which role the intracellular loop D plays in AQP0 function, we first tested the impact of p.D150H on protein location in HeLa cells. Intriguingly, unlike the wide type AQP0 which reached the plasma membrane, AQP0-D150H was trapped within the secretory pathway (mainly in the Golgi apparatus). Moreover, we confirmed the result with immunoblots in HEK-293T cells by extracting the membrane protein of transfected cells individually. When the cells were transfected with the same amount of plasmids, protein levels of AQP0-D150H were significantly lower than those of wide type AQP0 in membrane-enriched lysates, similar to previous results obtained in *Xenopus* oocyte expression systems [12]. Our data suggest that the mutation would impair the trafficking of AQP0 and hinder it from reaching the plasma membrane. However, we did not observe obvious cytotoxicity in the AQP0-D150H expressing cells as previous report [11] (data not shown).

Analysis of the AQP0 structure may shed light on the importance of aspartic acid at codon 150. The 3D crystal structure of AQP0 indicates that Asp-150 (negatively charged) interacts electrostatically with Arg-226 and Lys-228 (both positively charged) near the C terminus [29]. The p.D150H mutation results in the replacement of aspartic acid with positively charged histidine. This change of side chain is likely to disrupt the interaction between intracellular loop D and C terminus, leading to gross conformational changes by affecting the packing of peptide. As a consequence, misfolding of the mutant protein interfere with the correct trafficking to the plasma membrane.

In summary, this study reported a congenital progressive cortical punctate cataract caused by the p.D150H mutation of *MIP*. It is a new missense mutation which affects a highly conserved amino acid in the intracellular loop D of AQP0; no substitution in this domain has previously been associated with cataract. Moreover, this study presented the evidence that p.D150H damages the trafficking of AQP0 and causes the mutant protein not to reach the plasma membrane. However, since AQP0 has multiple functions, further studies focusing on the biological changes caused by p.D150H are needed.
